# The Mercapturomic Profile of Health and Non-Communicable Diseases

**DOI:** 10.3390/ht8020010

**Published:** 2019-04-23

**Authors:** Clara Gonçalves-Dias, Judit Morello, Valdir Semedo, M. João Correia, Nuno R. Coelho, Emilia C. Monteiro, Alexandra M. M. Antunes, Sofia A. Pereira

**Affiliations:** 1 CEDOC, Chronic Diseases Research Centre, NOVA Medical School, Faculdade de Ciências Médicas, Universidade NOVA de Lisboa, 1169-006 Lisboa, Portugal; claralgdias@gmail.com (C.G.-D.); valdir.semedo@nms.unl.pt (V.S.); mjoao.correia@nms.unl.pt (M.J.C.); nunofrcoelho@gmail.com (N.R.C.); emilia.monteiro@nms.unl.pt (E.C.M.); 2 Centro de Química Estrutural, Instituto Superior Técnico, ULisboa, 1049-001 Lisboa, Portugal; judit.morello@tecnico.ulisboa.pt (J.M.); alexandra.antunes@tecnico.ulisboa.pt (A.M.M.A.)

**Keywords:** mercapturate pathway, cysteine-*S*-conjugates, chronic inflammation, *N*-acetyl-transferase 8, cysteinyl-leukotrienes, dopamine, estrogen, disulfides, metabolomics, biomarkers

## Abstract

The mercapturate pathway is a unique metabolic circuitry that detoxifies electrophiles upon adducts formation with glutathione. Since its discovery over a century ago, most of the knowledge on the mercapturate pathway has been provided from biomonitoring studies on environmental exposure to toxicants. However, the mercapturate pathway-related metabolites that is formed in humans—the mercapturomic profile—in health and disease is yet to be established. In this paper, we put forward the hypothesis that these metabolites are key pathophysiologic factors behind the onset and development of non-communicable chronic inflammatory diseases. This review goes from the evidence in the formation of endogenous metabolites undergoing the mercapturate pathway to the methodologies for their assessment and their association with cancer and respiratory, neurologic and cardiometabolic diseases.

## 1. Brief Overview of the Mercapturate Pathway

**Figure 1:**
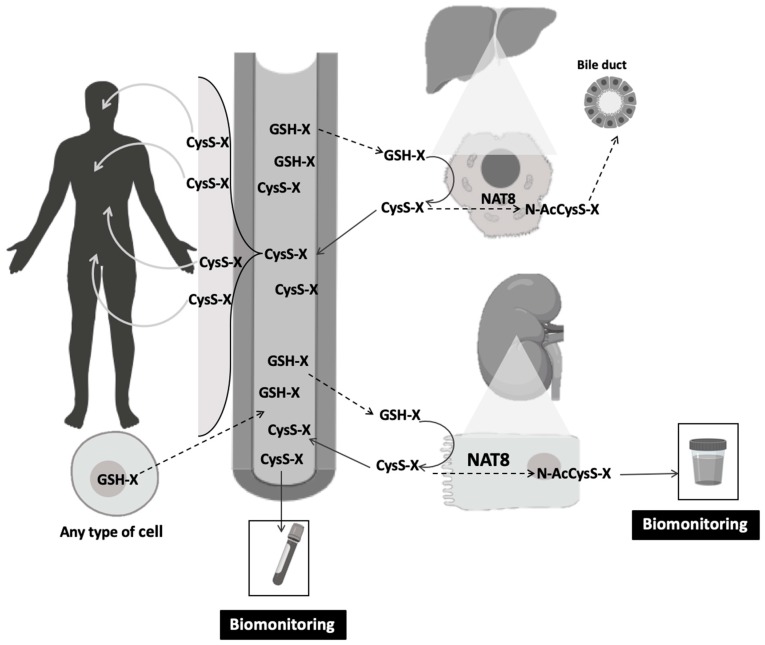
The mercapturomic profile of health and non-communicable chronic diseases. Any cell can generate GSH-*S*-conjugates that are excreted into the circulation and metabolized at the external apical membrane of kidney proximal tubular cells (major route) and hepatocytes (minor route). The Cys-*S*-conjugates that are formed might be subsequently detoxified by the N-acetyl-transferase NAT8, allowing the formation of mercapturates that are eliminated in urine. The Cys-*S*-conjugates can also be reabsorbed into the bloodstream and distributed into several organs. Blood and urine can be used for biomonitoring of mercapturate pathway-related metabolites. CysS-X: cysteine-*S*-conjugates; GSH-X: glutathione-*S*-conjugates; N-AcCysS-X: mercapturates; NAT8: *N*-acetyl-transferase 8.

The mercapturate pathway is one of the key traits of renal proximal tubular cells, although it is also present in hepatocytes [1]. The main function of this pathway is to detoxify electrophilic species [2]. These electrophiles might arise either from the metabolism of endogenous substances or from exogenous compounds (or their biotransformation products) present in air, food or water [3,4,5,6,7]. Once generated in any cell and upon conjugation with glutathione (GSH) (Figure 1), an electrophile-GSH-*S*-conjugate is formed [8]. As cells are not able to metabolize these conjugates intracellularly, those conjugates are effluxed into the bloodstream to undergo the mercapturate pathway. Thus GSH-*S*-conjugates are the precursors that will generate mercapturates, through the three sequential steps that constitute this pathway. The first two steps are extracellular and generate cysteinyl-glycine-*S*-conjugates (CysGly-*S*-conjugates) and cysteine-*S*-conjugates (Cys-*S*-conjugates) by the membrane-bound-enzymes, gamma-glutamyl-transferase (GGT) and dipeptidase or aminopeptidase-M, respectively [9,10,11]. Despite their presence in tissues such as liver, small intestine, lung, brain, spleen and pancreas, the main local of expression of these enzymes is the kidney tubule [12]. The Cys-*S*-conjugates enter the renal tubular cells and hepatocytes via various transporters including organic anion transport polypeptides and cystine/cysteine transporters for the last reaction of the mercapturate pathway [12,13,14]. The last step of this pathway relies on the microsomal *N*-acetyl-transferase 8 (NAT-8) that is expressed almost exclusively in the kidney proximal tubular cells, with much lower presence at the liver [1]. An *N*-acetyl-cysteine-*S*-conjugate, also known as a mercapturate, is lately formed upon NAT8 activity and is majorly eliminated in urine.

## 2. The Mercapturomic Profile

The metabolites that are formed through this pathway include the precursors GSH-*S*-conjugates and their catabolic products, the CysGly-*S*-conjugates and Cys-*S*-conjugates, and finally their mercapturates. This mercapturate pathway-related metabolites is herein called the mercapturomic profile. As Cys-*S*-conjugates seem to have significantly higher half-life than their precursors [4,15], they are the plausible ones to be used for biomonitoring purposes in human biological fluids (Table 1, Table 2, Table 3, Table 4 and Table 5). In addition, the urinary mercapturates represent a prominent non-invasive approach to profile this pathway. 

## 3. Biological Actions of Mercapturate Pathway-Related Metabolites

The effects of Cys-*S*-conjugates might have been underestimated, probably because the mercapturate pathway has been classically considered a detoxification route for xenobiotics. However, it is for instance known that the Cys-*S*-conjugate of cisplatin is more toxic to kidney tubular cells than cisplatin by itself [16]. Additionally, the Cys-*S*-conjugate of paracetamol is related to its nephrotoxicity, but not to its hepatotoxicity [17]. 

Cys-*S*-conjugates have been associated with hemodynamic properties [18], such as arteriolar vasoconstriction [19,20,21] and enhanced postcapillary venules permeability [22]. Cys-*S*-conjugates are involved in glucose-stimulated insulin secretion [23] and might have pro-inflammatory [5], cytotoxic [16,17,24,25], genotoxic [25] and immunogenic [26] properties. Most of available studies have investigated the role of specific mercapturate pathway related metabolites in an experimental model or in a particular group of patients. Thus far, no work has given a comprehensive view of the mercapturomic profile, similarly to what it is performed for protein adducts [27]. In fact, Wang and Ballatori (1998) have brilliantly reviewed dozens of compounds that generate GSH-*S*-conjugates [28] such as leukotrienes [3], prostaglandins [29] and lipid peroxidation products [6,7].

The cysteinyl-leukotrienes (CysLTs) might be the best described example in the literature, concerning its association with non-communicable diseases. CysLTs are products of arachidonic acid metabolism and key mediators of inflammatory conditions [30,31,32] and stem from the catabolism of leukotriene C4 (LTC4), which is a GSH-*S*-conjugate. Extracellular LTC4 undergoes a two-step catabolic process originating the CysGly-*S*-conjugate (leukotriene D4, LTD4) and Cys-*S*-conjugate (leukotriene E4, LTE4) respectively, through the mercapturate pathway [31,32]. These compounds are generally termed CysLTs, although this denomination fully suits only LTE4, which has the longest half-life [15]. LTE4 mercapturate formation is mediated by NAT8 activity as described by the team of Veiga da Cunha (2010) [3]. CysTL are well known for their role in the pathophysiology of asthma and increasing evidence links these metabolites with non-communicable chronic inflammatory conditions [33,34], namely cardiovascular, neurologic and kidney disease [35] and cancer [36,37]. Altogether, non-communicable diseases represent the most common cause of death and multi-morbidity in the modern world [38]. Expanding investigations have shown that many of these diseases share pathophysiological mechanisms, with a similar profile of molecular changes, despite affecting diverse organs and systems differently. To fulfil this concept in a mercapturate pathway-related perspective, we herein review the available knowledge about the association between mercapturate pathway-related metabolites and the major non-communicable diseases. All included reports are clinical studies. 

## 4. The Human Mercapturomic Profile in Health and Disease

### 4.1. Respiratory Diseases

CysLTs are important inflammatory mediators in the pathophysiology of respiratory disorders (Table 1) [39,40,41,42]. They are potent bronchoconstrictors and can cause acute and chronic structural defects in the airways [43,44,45]. Common treatment of asthma might include CysLTs receptor type 1 antagonists. There are also inhibitors available for 5-lypoxygenase, the enzyme involved in the synthesis of the precursor of CysLT from arachidonic acid. Three studies evaluated CysLTs in saliva, exhaled breath condensate and urine samples of patients with asthma (Table 1). Both chronic and acute asthma were associated with increased levels of LTE4 in all the biological matrixes analyzed [40,46,47]. Moreover, smoking habits did not affect LTE4 levels in exhaled breath condensate and the use of CysLTs receptor antagonists during asthma exacerbation did not affect LTE4 levels in urine [40]. Cys-*S*-conjugates which are disulfides were increased in children with difficult-to-treat asthma [34] and associated with asthma severity, including poorer control of symptomatology, greater medication use and a worse response to glucocorticoid therapy [48]. 

CysLTs have also been associated to silica-induced lung fibrogenesis [49]. In fact, increased LTE4 levels were observed in exhaled breath condensate of patients with pneumoconiosis derived from asbestos and silica exposure [39]. 

### 4.2. Cancer

Cys-*S*-conjugates have also been described in different types of cancer, namely in melanoma, non-Hodgkin lymphoma, breast, ovarian and thyroid cancer (Table 2).

Melanoma was linked to the melanin metabolite 5-*S*-Cys-DOPA (Cys-DOPA). In melanocytes, the amino acid L-DOPA is oxidized into a highly reactive dopaquinone that after binding to a sulfhydryl donor as glutathione is further oxidized to pheomelanin, a yellow to reddish form of melanin. Increases in serum Cys-DOPA have been associated with poor prognosis of malignant melanoma and shorter survival times [50,51,52,53,54]. Additionally, Cys-DOPA also increased in melanoma recurrence after chemotherapy or surgery [50,53,54].

Estrogen metabolism is strongly implicated in the development of hormonal cancers [55,56,57]. Estrogen metabolites, namely 2- and 4-hydroxyestrone and 2- and 4-hydroxyestradiol might generate electrophilic metabolites, and for mercapturate pathway-related metabolites their urinary levels were found to be decreased in patients with breast cancer or non-Hodgkin lymphoma relative to healthy subjects [58,59,60]. Additionally, the ratio of depurinating estrogen deoxyribonucleic acid (DNA) adducts to estrogen metabolites and conjugates (including GSH-*S*-conjugates, Cys-*S*-conjugates and mercapturates) was higher in cases of thyroid and ovarian cancer in comparison with healthy individuals [56,57]. Changes in Cys-*S*-conjugates that are disulfides were also observed in leukemia, lymphoma and colorectal adenoma [61,62].

### 4.3. Neurologic Diseases

Parkinson’s disease (PD) is characterized by severe depletion of dopamine (DA) [63]. The role of dopamine related cysteinyl-*S*-conjugates in PD has been investigated in order to evaluate how the failure of anti-oxidative mechanisms, in the prevention of spontaneous dopamine oxidation, might contribute the degeneration of dopaminergic neurons (Table 3). 

Dopamine can be oxidized following non-enzymatic and enzymatic pathways. Dopamine can spontaneously oxidize to dopamine-o-quinone, which forms conjugates GSH-*S*-conjugates. Dopamine can also be oxidized by monoamine oxidase to 3,4-dihydroxyphenylacetaldehyde, which is further metabolized by aldehyde dehydrogenase to 3,4-dihydrophenylacetic acid (DOPAC) and then into homovanillic acid upon catechol-O-methyltransferase activity [64,65].

In 1989, Fornstedt and colleagues [66] identified 5-Cys-*S*-conjugates of DOPA, DA and DOPAC in three brain regions (substantia nigra, putamen and caudate nucleus) of post-mortem brains from patients with and without depigmentation and neuronal loss within the substantia nigra. The levels of DOPA, DA and DOPAC were decreased in the depigmented group.

Additionally, while no differences were found for the Cys-*S*-conjugates, the authors observed an increase in the ratio of Cys-DA/DA and Cys-DOPAC/DOPAC in the substantia nigra and Cys-DOPA/DOPA in the putamen of the depigmented group [66]. Similar results were later obtained with patients with PD and parkinsonism (PD and multiple system atrophy parkinsonism). Importantly, patients were not on DOPA therapy. The levels of Cys-DA were not affected in patients with parkinsonism. Nevertheless, as DOPAC or homovanillic acid were decreased, both Cys-DA/DOPAC or Cys-DA/homovanillic acid ratios were increased in these patients [67,68]. The work of Goldstein and co-authors [67] also showed that Cys-DA and DOPAC have the same source: the cytoplasmic dopamine. Thus, the dopamine denervation associated with parkinsonism would be expected to produce equal proportional decreases in Cys-DA and DOPAC levels and consequently unchanged Cys-DA/DOPAC ratios. The authors were not able to explain the observed decrease in DOPAC without the decrease in Cys-DA [67]. Even though, the authors suggested that this might be due to decreased antioxidant capacity [69] and aldehyde dehydrogenase activity [70]. Interestingly, substantia nigra of PD patients has a 50% reduction of their GSH levels [71,72]. This decrease can be presumably due to the reaction of GSH with DA semiquinones or quinones [73]. At the same time, decreased antioxidant capacity might shift the balance from dopamine to dopamine quinone and finally to Cys-DA, which will explain the absence of decreased levels Cys-DA. In opposition, there is one study reporting increased levels of 5-S-Cys-conjugates of DOPA, DA and DOPAC at substantia nigra of patients with PD. However, all patients were under L-DOPA treatment, which could have influenced the results [74].

Catechol estrogens are also present in the brain and, like dopamine, can be bioactivated to catechol quinones able to form adducts with GSH and undergo the mercapturate pathway for elimination. Urinary estrogen-catechol Cys-*S*-conjugates were lower and estrogen-DNA adducts were higher in PD patients than in healthy controls [75]. The authors suggested that there is an unbalanced estrogen metabolism in PD and that the protective pathways might be unable to avoid the oxidation of catechol estrogens and further DNA adducts formation. 

On the other hand, neuro-inflammation might also play a role in autism [76,77]. The levels of CysLTs have been investigated in autistic children, together with a sensitive indicator of bioactive products of lipid peroxidation and oxidative stress, the 8-isoprostane [78,79]. The authors proposed both CysLTs and 8-isoprostane as markers for early recognition of sensory dysfunction in autistic patients that might facilitate earlier interventions [78].

CysLTs increases at the central nervous system [80,81,82], might also be involved in edema formation in brain tumor patients [83].

### 4.4. Cardiometabolic Diseases

There are several works reporting the association of CysLTs in cardiometabolic diseases (Table 4) and different mechanisms might explain this association. For instance, in cardiometabolic diseases, the 5-lipoxygenase pathway that contributes to CysLTs formation is activated, the CysLTs receptors (mainly CysLT2R) are strongly expressed in cardiac, endothelial and vascular smooth muscle cells. CysLTs exert negative inotropic action on the myocardium and mediate coronary vasoconstriction [84]. Moreover, CysLTs may have pro-atherogenic effects; they may stimulate proliferation and migration of arterial smooth muscle cells and platelet activation [36]. 

Winking and collaborators (1998) measured urinary LTs in patients suffering from spontaneous intracerebral hemorrhage. Urinary LTC4, LTD4 and LTE4 levels were positively associated with hematoma volume and decreased after hematoma removal by surgery [85].

Regarding coronary artery diseases, urinary LTE4 levels were increased in patients admitted in the hospital with acute chest pain derived from acute myocardial infarction and unstable angina compared with controls [86]. Likewise, urinary LTE4 levels were higher in patients with chronic stable angina than controls before surgery [87]. In another study, urine and plasma levels of CysLTs increased during, and after, cardiac surgery with cardiopulmonary bypass. Interestingly, that increment was greater in patients with moderate-to-severe chronic obstructive pulmonary disease than in patients without this condition [88]. The authors hypothesize that these differences may be related to neutrophil activation and higher lung and airway production of CysLTs in patients with chronic obstructive pulmonary disease.

CysTL were also evaluated in individuals with atherosclerosis lesions in the carotid artery concomitantly with or without periodontal disease. This study was motivated by several reports that have been associating periodontal disease with the development of early atherosclerosis and increased risk of myocardial infarction [89,90,91]. The sum of LTC4, LTD4 and LTE4 was increased in gingival crevicular fluid in subjects with higher dental plaque and also in subjects with atherosclerotic plaques in the carotid artery, regardless of periodontal status [92]. 

CysLTs might play a role in the development of the cardiovascular complications associated with obstructive sleep apnea (OSA). Urinary LTE4 levels were associated with obesity and hypoxia severity in patients diagnosed with OSA. Continuous positive air treatment decreased LTE4 by 22% only in OSA patients with normal body max index (BMI). Additionally, LTE4 levels were higher in non-obese OSA patients vs. matched controls [93]. In another study, Gautier-Veyret and co-authors (2018) found that urinary LTE4 levels were independently associated with age, history of cardiovascular events and severity of hypoxia in patients with OSA with and without previous cardiovascular events. As such, LTE4 levels were higher in OSA patients with no previous cardiovascular events than in controls with no previous cardiovascular events. Urinary LTE4 levels were also associated with intima-media thickness, suggesting the activation of CysLTs pathway as a driver of vascular remodeling in OSA [94]. 

CysLTs were also evaluated in patients with diabetes. Urinary LTE4 levels were higher in patients with type 1 diabetes than in controls [95] and decreased 32% after intensive insulin treatment [96]. These results suggest that hyperglycemia activates arachidonic acid metabolism and consequent CysLTs formation. Interestingly, glucose can also generate Cys-*S*-conjugates that are far more stable than glucose-GSH. In specific, higher urinary levels of glucose-Cys were detected in patients with diabetes [4].

Cys-*S*-conjugates that are disulfides were related with hypertension, diabetes and Framingham risk score in coronary heart disease patient [97,98] as well as impaired microvascular function and greater epicardial necrotic core [97]. Moreover, these conjugates and GSH-Cys-*S*-conjugates were independent predictors of endothelium-dependent vasodilation [97].

## 5. Methods in Mercapturates Profiling

Mercapturate pathway-related metabolites and their profile might be useful as biomarkers in characterizing human exposure to electrophilic endogenous substrates and its relation to health and disease. The methodological strategies herein reviewed for the determination of mercapturate pathway-related metabolites are presented in Table 5. These compounds have been measured in different human fluids and tissues requiring pre-treatment of samples. The studies herein reviewed quantify only one type or family of mercapturate pathway-related metabolites (dopamine, estrogens, cysteinyl-leukotrienes and cysteinyl-*S*-conjugates which are disulfides). Those metabolites were quantified by different methodologies including liquid chromatography with ultraviolet detector or fluorescence detector or mass spectrometry detector, enzyme-linked immunosorbent assay and radioimmunoassay (Table 5).

## 6. Trends and Limitations

**Figure 2:**
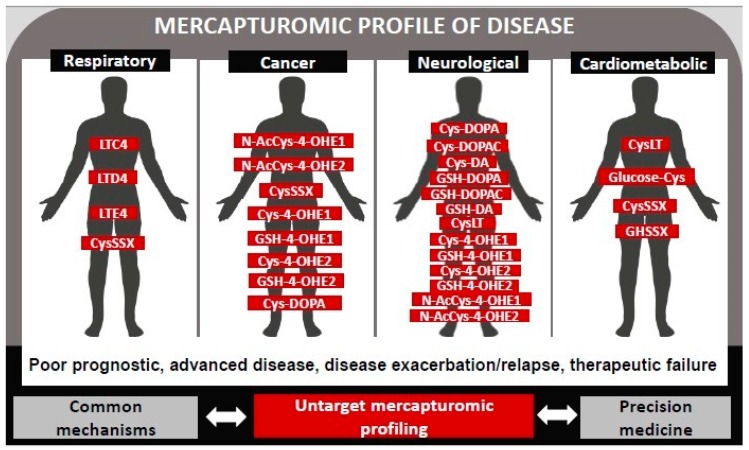
Mercapturomic profile of disease. This profile was defined by reviewing the mercapturate-pathway related metabolites that have already been associated with non-communicable diseases (prognostic, progression, therapeutic response) in clinical studies.

Herein we review the clinical studies that reported associations between, one of or a family of, mercapturate pathway-related metabolites with a particular disease. In fact, most of available evidence on the association of the mercapturomic profile with health and disease has been obtained by a targeted approach (Figure 2). Future work might focus on a comprehensive qualitative and quantitative analysis of the totality of mercapturate-pathway related metabolites, in similarity to what has been done for protein addutomics [104].

One of the main limitations to assess the global mercapturomic profile is the fact that the mercapturate pathway-related metabolites are often minor metabolites [105]. Despite the enormous technological advances in MS instrumentation, the identification of this minor adducts is still challenging. New approaches are needed for providing accuracy and sensitivity along with quantitative information. The future obstacles will involve not only sample pre-treatment procedures, but also optimization of MS and data analysis strategies. 

On the other hand, in vivo models of disease will allow to investigate the origin and metabolism of these compounds as well as their distribution in the body. In fact, these compounds have been described to be found in several matrices, including tissues, urine, plasma, exhaled breath condensate, saliva, polymorphic blood mononuclear cells or gingival crevicular fluid that might require different pre-treatment procedures. 

## 7. Innovative Potential

Many chronic diseases with an inflammatory component display significantly increased levels of electrophiles. The mercapturomic profile might represent a useful tool to globally characterize both environmental and internal electrophile exposomes and its relation to disease (Figure 2). This holistic omic-approach is expected to provide unique information that includes the identification of new therapeutic targets and commonalities related to mechanisms of different diseases that might facilitate therapeutics development and define preventive strategies. Additionally, this approach might constitute an effective tool to define the mercapturomic phenotypes of drug resistance and adverse reactions; disease progression, encouraging precision medicine standards. Finally, as many environmental compounds undergo this pathway it will also contribute to a better understanding of the contribution of environment to non-communicable diseases.

## Figures and Tables

**Table 1:** Mercapturomic profile of respiratory diseases.

Disease	Aim	Study Population	Mercapturomic Profile
**Pneumo-coniosis**	Evaluate the impact of pneumoconiosis and systemic diseases, drugs and diet on LTC4 and LTE4 levels measured in EBC, plasma and urine Ref [39]	A total of 82 patients with pneumoconiosis: 45 from asbestos exposure (mean age 70 yo; 53% men) 37 from silica exposure (mean age 69 yo; 97% men) and 27 CTLs Subjects with systemic disorders (atherosclerosis, cancer) were present in all groups	In CTLs, plasma LTE4 correlated with nephrolithiasis (+) and fibrates (+) In asbestosis, CysLTs correlated with lung function (−), plasma LTC4 correlated with steroids (+) In silicosis, urine LTD4 correlated with kidney failure (+), and salicylates (+); and plasma LTE4 with vitamin C and E (+) EBC: LTB4 asbestosis > silicosis and CTL; LTD4 and LTE4 asbestosis and silicosis > CTL. Urine: LTD4 asbestosis > CTL. Plasma: LTE4 asbestosis > CTL
**Asthma**	Evaluate the effect of smoking in LTD4 and LTE4 levels in asthma Ref [40]	EBC from 59 asthmatic patients: 30 smokers (mean age 34 yo; 50% men) and 29 non-smokers (mean age 34 yo; 48% men); and 29 CTLs (mean age 34 yo; 48% men; non-smokers)	LTD4 asthmatic smokers > asthmatic non-smokers and CTLs LTE4 asthmatic > CTL LTE4 correlated with FEV1/FVC ratio (−)
	LTE4 levels in treatment of asthma exacerbation with CysLTR1 antagonists Ref [46]	184 patients with acute asthma at ED (age 35 yo): 123 on ß agonist + montelukast; 61 on ß agonist + placebo. Sampling at ED and 2 weeks after	Urine: LTE4 during exacerbations > 2 weeks later. No differences in LTE4 during exacerbation or 2 weeks later in patients receiving montelukast or placebo. LTE4 correlated with FEV_1_ (−) during exacerbation and 2 weeks later
	CysLTs levels in saliva of AIA patients Ref [47]	26 non-smoking asthmatic patients: 15 AIA (mean age 51 yo; 40% men) and 11 ATA (mean age 55 yo; 36% men); 10 CTLs; patients were also divided in mild (*n* = 6) and severe (*n* = 9) asthma	Saliva LTC4 and LTE4 AIA > ATA and CTL Urine LTE4 AIA > ATA and CTL and LTE4 severe AIA > mild AIA
	Characterize systemic Cys-*S*-conjugates that are disulfides and its association with asthma Ref [48]	Plasma and PBMCs samples from 99 children with asthma (median age 12 yo; 67% men) and 15 CTLs (median age 10 yo; 20% men). On treatment for asthma, 57 difficult-to-treat children underwent glucocorticoid responsiveness test	Plasma: CysSSCys and E_h_CysSH/CysSSCys in asthma > CTL. PBMCs: GSSG in asthma > CTL In glucocorticoid treated children, E_h_CysSH/CysSSCys in non-responders > responders before and after treatment

(−): negative correlation; (+): positive correlation; AIA: aspirin-intolerant asthma; ATA: aspirin-tolerant asthma; Cys: cysteine; CysSH: free cysteine; CysLTs: cysteinyl-leukotrienes; CysSSCys: cystine; CysSSG: cysteine-glutathione disulfide; EBC: exhaled breath condensate; ED: emergency department; E_h_: redox potential; FEV_1_: forced expiratory volume in 1 second; FEV1/FVC ratio: ratio of forced expiratory volume in one second by forced vital capacity; GSSG: oxidized glutathione; LTC4: leukotriene C4; LTD4: leukotriene D4; LTE4: leukotriene E4; PBMCs: peripheral blood mononuclear cells; yo: years old.

**Table 2:** Mercapturomic profile of cancer.

Disease	Aim	Study Population	Mercapturomic Profile
**Melanoma**	Usefulness of serum Cys-DOPA levels in MM prognosis and response to immuno-chemotherapy Ref [50]	Serum samples from 11 patients with MM before and after each immunochemotherapy cycle Mean age 47 yo; 64% men	Cys-DOPA MM patients after therapy > MM patients before therapy > healthy controls Patients with declines in Cys-DOPA > 68% of treatment cycles had longer survival time
	Value of Cys-DOPA in different stages of MM Ref [51]	Serum samples from 252 patients followed for 1 to 4 months: patients with no evidence of MM after surgery or chemotherapy (asymptomatic patients), patients with MM classified according to symptoms or clinical I-III stages. Range age 18–86 yo; 51% men	Cys-DOPA symptomatic > asymptomatic patients Cys-DOPA stage III > stage I and II patients Cys-DOPA stage III > primary tumor, lymph node and lung metastasis symptoms ↑ Cys-DOPA correlated with tumor mass
	Usefulness of serum Cys-DOPA in melanoma progression and prognosis Ref [52]	Serum samples of 218 melanoma patients Mean age 55 yo; 51% men	Cys-DOPA > 10 nmol/L in stage IV patients Elevation of Cys-DOPA preceded or occurred at the same time of clinical detection of visceral metastasis in, respectively, 33% and 37% of cases ↑ Cys-DOPA associated with shorter survival time
	Case report for diagnosis of rectal malignant melanoma with Cys-DOPA Ref [53]	Serum samples of a woman 84 yo diagnosed with rectal MM	Before tumor resection: Cys-DOPA = 26 nM. Three months after surgery: Cys-DOPA = 12.6 nM (still > 10 nM) and CT scan confirmed multiple liver and lung metastasis.
	Usefulness of serum Cys-DOPA as a biomarker for prognosis and early detection of relapse of malignant melanoma Ref [54]	Serum samples of 120 patients with advanced stage malignant melanoma Mean age 64 yo; 41% men	Cys-DOPA in advanced stages (III and IV) > early stages (0–II) patients. In patients with advanced stages, Cys-DOPA > 15 nM correlated with a poor prognosis In 11/14 patients with melanoma recurrence, Cys-DOPA > 10 nM around the time of relapse
**Colorectal adenoma**	Evaluate the effects of antioxidant micronutrients on oxidative and inflammatory biomarkers in sporadic colorectal adenoma. Ref [62]	Pilot, randomized, double-blind, placebo-controlled clinical trial. Plasma samples from 47 patients with a history of sporadic colorectal adenoma: 23 under placebo (median age 59 yo; 52% men) and 24 under antioxidant treatment with 800 mg vitamin E, 24 mg β-carotene, 1000 mg vitamin C, 200 μg L-selenomethionine, 7.2 mg riboflavin, 80 mg niacin, 60 mg zinc and 5 mg manganese (median age 61 yo; 50% men).	CysSSCys in the antioxidant (−39%) < placebo group In the antioxidant group, ↓ CysSSCys was only statistically significant in nonsmokers (−35%) vs. smokers (−12%)
**Leukemia lymphoma**	Determine the effect of high-dose chemotherapy and type of parenteral nutrition in circulating antioxidants in patients undergoing BMT Ref [61]	Double-blind, controlled, randomized clinical trial. Plasma samples from 24 BMT patients (mean age 40 yo; 58% men) with NHL (*n* = 10), chronic myeloid leukemia (*n* = 8), Hodgkin disease (*n* = 4), acute myeloid leukemia (*n* = 1) and T cell lymphoma (*n* = 1). Most patients received chemotherapy or chemotherapy + radiation. Patients were divided into treatment with standard parenteral nutrition with amino acids, dextrose, lipids, vitamins minerals and electrolytes (mean age 41 yo; 36% men) or modified parenteral nutrition (mean age 38 yo; 69% men) with electrolytes, vitamins, minerals and less lipids. Samples were collected before chemotherapy and BMT (baseline) and 1, 3, 7, 10 and 14 days after BMT.	↑ E_h_GSH/GSSG, E_h_Cys/CysSSCys and CysSSCys over time, regardless of treatment or parental nutrition type
**non-Hodgkin lymphoma**	Determine if the estrogen metabolites and adducts are involved in the etiology of NHL Ref [60]	Urine samples from 15 NHL patients (median age 59 yo; 100% men) and 30 CTLs (median age 60; 100% men). Estrogen metabolites included Cys, GSH and NAC conjugates of 4-OHE1(E2) and 2-OHE1(E2).	Cys, GSH and NAC conjugates of 4-OHE1(E2) in CTL > NHL group
**Thyroid cancer**	Investigate the role of estrogen metabolites and adducts in thyroid cancer Ref [57]	Urine samples from 40 women with thyroid cancer (mean age 47 yo) and 40 CTL women (mean age 47 yo). The estrogen metabolites included Cys, GSH and NAC conjugates of 4-OHE_1_(E_2_) and 2-OHE_1_(E_2_).	Ratio depurinating estrogen-DNA adducts to estrogen metabolites and *S*-conjugates in thyroid cancer > CTL group
**Ovarian cancer**	Investigate the role of estrogen metabolites and adducts in ovarian cancer Ref [56]	Urine samples from 33 women with ovarian cancer (mean age 58 yo) and 34 CTL women (mean age 58 yo). The ratio of depurinating estrogen DNA adducts to estrogen metabolites and *S*-conjugates was obtained. The estrogen metabolites included Cys, GSH and NAC conjugates of 4-OHE_1_(E_2_) and 2-OHE_1_(E_2_).	Ratio depurinating estrogen DNA adducts to estrogen metabolites and *S*-conjugates in ovarian cancer > CTL.
**Breast cancer**	Evaluate the urinary levels of estrogen metabolites and adducts in breast cancer	Urine samples from 12 women with high-risk for breast cancer (mean age 52 yo), 17 with breast cancer (mean age 54 yo) and 46 CTLs (mean age 50 yo). The ratio of depurinating estrogen DNA adducts to metabolites was obtained. Estrogen metabolites included Cys, GSH and NAC conjugates of 4-OHE1(E2) and 2-OHE1(E2). Ref [58]	Cys, GSH and NAC conjugates of 2-OHE_1_(E_2_) in CTL > other groups.
		Urine samples from 40 women with high-risk for breast cancer (median age 57 yo); 40 with newly diagnosed breast cancer (median age 58 yo); CTLs (median age 45 yo). All without estrogen-containing treatments. The ratio of depurinating estrogen DNA adducts to metabolites was obtained. Estrogen metabolites included Cys, GSH and NAC conjugates of 4-OHE1(E2) and 2-OHE1(E2). Ref [59]	Cys, GSH and NAC conjugates of 2-OHE1(E2) and 4-OHE1(E2) in CTL > other groups. Ratio in breast cancer and high-risk group for breast cancer > in CTL.

↑: higher; ↓: lower; 2-OHE_1_(E_2_), 2-hydroxyestrone(estradiol); 4-OHE_1_(E_2_), 4-hydroxyestrone(estradiol); Cys-DOPA: 5-*S*-Cysteinyl-dopa; BMT: bone marrow transplantation; CTL: controls; Cys: cysteine; CysSSCys: cystine; E_h_: redox potential; GSH: glutathione; GSSG: oxidized glutathione; MM: malignant melanoma; NHL: non-Hodgkin lymphoma; yo: years old.

**Table 3:** Mercapturomic profile of neurologic diseases.

Disease	Aim	Study Population	Mercapturomic Profile
**Parkinson**	Investigate the association between SN’s degenerative changes and the occurrence of Cys-DOPA, Cys-DA and Cys-DOPAC Ref [66]	Postmortem brain samples (SN, PUT and CN sections) from 17 individuals. 72–90 yo; 41% men. Samples were divided according to degree of depigmentation and neuronal loss within SN (12 pigmented, 5 depigmented)	DOPA, DA, DOPAC depigmented < pigmented in SN. No differences for Cys-*S*-conjugates Cys-DA/DA, Cys-DOPAC/DOPAC depigmented > pigmented in SN Cys-DOPA/DOPA depigmented > pigmented in PUT
	Evaluate the levels of Cys-DA and HVA in CSF samples of PD patients Ref [68]	CSF samples from 20 PD patients (mean age 69 yo, 85% men) and 16 CTLs (mean age 60 yo years, 63 % men); Samples under and 5 days after L-DOPA withdrawal	HVA in PD patients after L-DOPA withdrawal < CTLs. No differences for Cys-DA among groups. Cys-DA/HVA in PD after L-DOPA withdrawal > CTLs.
	Assess Cys- and GSH-conjugates of DA, DOPA and DOPAC in brain tissue and changes on their levels in PD Ref [74]	Postmortem brain samples from six PD patients with PD (mean age 77 yo, L-DOPA therapy) and six CTLs (mean age 81 yo); Brains were dissected into 11 regions	Detectable conjugates in most brain regions, with higher levels in SN and PUT GSH-conjugates < Cys-conjugates Cys-conjugates in SN in PD > CTLs
	Assess estrogen metabolites and adducts in PD Ref [75]	Urine samples from 20 PD patients (mean age 62 yo; 75% men; all under levodopa) and 40 CTLs (mean age 63 yo; 75% men).Estrogen metabolites included Cys-, GSH- and NAC- conjugates of 4-OHE_1_(E_2_) and 2-OHE_1_(E_2_)	Cys, GSH and NAC conjugates of 4-OHE_1_(E_2_) CTL > PD group
	Assess the value of Cys-DA/DOPAC ratio in CSF as a specific biomarker of parkinsonism Ref [67]	CSF samples from 24 PD patients (mean age 61 yo, 58% men); 32 MSA-P (mean age 60 yo; 66% men); 18 PAF (mean age 63 yo; 67% men) and 32 CTLs (mean age 53 yo; 53% men). Patients were not on levodopa or MAO inhibitors	Cys-DA levels were similar among groups; Cys-DA/DOPAC > 2-fold in PD and MSA-P than PAF and CTL groups Cys-DA/DOPAC was correlated with putamen/occipital ratios (−) and washout fractions of ^18^F-fluorodopa-derived radioactivity (+)
**Autism**	Determine the correlation of 8-isoprostane, LTs, age and autism severity scales Ref [78]	Plasma samples from 44 autistic children (mean age 7 yo) and 40 CTLs (mean age 7 yo). Autistic cases were all simple and tested negative for the fragile X gene mutations *LTs measured = LTA4 + LTC4 + LTD4 + LTE4	CysLTs and 8-isoprostane in autistic > CTL. CysLTs correlated with 8-isoprostane (+) SSP test correlated with CysLTs and 8-isoprostane (−)

(−): negative correlation; (+): positive correlation; 2-OHE_1_(E_2_), 2-hydroxyestrone(estradiol); 4-OHE_1_(E_2_), 4-hydroxyestrone(estradiol); Cys-DOPA: 5-*S*-Cysteinyl-dopa; Cys-DA: 5-*S*-Cysteinyl-dopamine; CT: CTL: controls; CSF: cerebrospinal fluid; DA: dopamine; DOPA; L-3,4-dihydroxyphenylalanine; CN: caudate nucleus; DOPAC; 3,4-dihydroxyphenylacetic acid; GSH: glutathione; HVA: homovanillic acid; LT: leukotrienes; LTB4: leukotriene B4; LTC4: leukotriene C4; LTD4: leukotriene D4; LTE4: leukotriene E4; MAO: Monoamine oxidase; MSA-P: parkinsonian multiple system atrophy; PAF: pure autonomic failure; PD: Parkinson’s disease; PUT: putamen; SI: substantia innominata; SN: substantia nigra; yo: years old. SSP Short Sensory Profile.

**Table 4:** Mercapturomic profile of cardiometabolic diseases.

Disease	Aim	Study Population	Mercapturomic Profile
**Diabetes**	To evaluate the influence of diabetes, glycaemia control and ACE inhibitor on LTE4 excretion. Ref [95]	Urine samples from 34 T1D patients: 20 with good metabolic control (age 39 yo; 55% men), 14 poor metabolic control (age 41 yo; 50% men); 28 CTLs (age 39 yo; 43% men). All nonsmokers	LTE4 T1D > CTL. LTE4 in T1D with poor metabolic control > CTL No influence of ACE Inhibitors on urinary LTE4
	Evaluate the effect of insulin treatment on the urinary excretion of LTE4. Ref [96]	Urine samples from 20 T1D (mean age 37 yo; 35% men) and 19 T2D patients (mean age 58 yo; 68% men). Non-smokers. Intensive insulin treatment over 3 months	↓ LTE4 after insulin treatment (−32%) in T1D but not in T2DM
	Assess if Cys is a good transglycating agent. Ref [4]	Urine samples from five diabetic patients and two normoglycemic subjects	Glucose-Cys diabetes > normoglycemic subjects
	To evaluate the association of urinary LTE4 with endothelial function Ref [99]	Urine samples from 30 (median age 65 yo; 80% men) T2DM subjects of at least 2 years duration and eGFR 71 (14–129) mL/min	Decreased renal function associated with ↓ urinary LTE4; LTE4 associated with serum creatinine (−) and eGFR (+); eGFR was an independent predictor of urinary LTE4 levels
**OSA-related atherogenesis**	Identify the factors influencing LTE4 levels and the role of LTE4 in OSA-related atherosclerosis Ref [94]	Urine samples from 170 OSA patients (mean age 57 yo; 81 % men): 136 CVE free and 34 with previous CVE; 29 CTLs (mean age 52 yo; 52% men): 22 CVE free and seven with previous CVE	LTE4 associated with age, min SaO_2_ and history of CVE and intima-media thickness. LTE4 in OSA CVE free patients > CTL CVE free group. Increase related to minSaO_2_ and traditional risk factors of the 10-year CDV risk score
**OSA-related obesity**	To evaluate the influence of obesity and CPAP in urinary LTE4 levels and the role of LTE4 as biomarker of inflammation in patients with OSA. Ref [93]	Urine samples from 40 non-obese OSA patients (mean age 49 yo; 85% men) and 25 CTLs (mean age 45 yo; 72% men). A group of 72 OSA patients with any BMI starting CPAP (mean age 51 yo; 81% men) was included to study confounder factors of LTE4. All nonsmokers	LTE4 in non-obese OSA > CTLs. In the 40 non-obese OSA patients, LTE4 was correlated with % of time spent with SaO_2_ < 90% (+). In the 72 OSA patients, BMI and % of time spent with SaO_2_ < 90% were identified as independent predictors of LTE4. CPAP treatment for at least 4 weeks ↓ LTE4 by 22% only in OSA patients with normal BMI
**Intra-Cerebral hemorrhage**	Quantify LTs in urine of spontaneous ICH patients and evaluate its impact in the edema formation. Ref [85]	Urine samples from 17 spontaneous ICH patients (mean age 58 yo; 53% men): 12 treated surgically and five conservatively. Sampling before treatment and during the five following days. CysLTs measured = sum of LTC4, LTD4 and LTE4	CysLTs correlated with hematoma volume (+) CysLTs 5 days after < before surgery CysLTs did not decrease after the conservative treatment
**Coronary artery diseases**	Assess LTE4 during and after acute coronary syndromes Ref [86]	Urine samples from 16 AMI (mean age 51 yo; 88% men); 14 UA patients (mean age 52 yo; 21% men); eight clinical CTLs (non-ischemic heart pain) (88% mean) and 10 normal CTLs (50% men) CTLs (non-evidence of coronary artery disease). Samples were collected upon admission with acute chest pain and 3 days after	LTE4 in MIA and UA at admission > CTL groups LTE4 on admission > 3 days after UA
	Study the relation between the systemic LTE4 levels and stable coronary artery disease before and after bypass surgery Ref [87]	Urine samples from 13 chronic stable angina (mean age 59 yo; 100% men) and 12 CTLs (mean age 44 yo; 100% men). Single urine patients from CTLs and patients before surgery; postoperative 24 h urine samples over seven successive days. 6/13 patients on aspirin until a few days before surgery. All but three patients on aspirin after the operation.	LTE4 in preoperative patients > CTL LTE4 2 days after > before surgery
	Detect the formation of CysLTs and atherosclerosis lesions in carotid artery in subjects with and without periodontitis Ref [92]	GCF samples from 19 subjects with periodontitis (mean age 55 yo; 63% men; 13 with atherosclerotic plaques in carotid artery) and 16 CTLs (mean age 53 yo; 44% men; five with atherosclerotic plaques in carotid artery).	Subjects with atherosclerotic plaques in periodontitis > in CTL CysLT higher in periodontitis with higher dental plaque index CysLTs in subjects with > subjects without atherosclerotic plaques in all subjects independently on periodontitis
**Coronary artery diseases**	Assess oxidative stress parameters in the bloodstream as a reliable predictor of endothelial function. Ref [98]	Plasma samples from 124 healthy nonsmokers without CDV risk factors (44 yo; 40% men). At recruitment, 41 patients were HTN, diabetes or BMI ≥ 30. Vasodilation was measured at the brachial artery	CysSSCys related with age(+), BMI(+), HTN(+) Framingham score CysSSCys in HTN, diabetes or BMI ≥ 30 > remaining individuals CysSSG related with TG(−), HDL(+), HTN(+). CysSSCys and CysSSG independent predictors of endothelium-dependent vasodilation
	Test if ↑ oxidative stress was associated with impaired coronary microvascular function and plaque necrotic core content Ref [97]	Plasma samples from 47 patients with an abnormal non-invasive stress test, stable angina or stabilized acute coronary syndrome undergoing cardiac catheterization (mean age 58 yo; 64% men). Microvascular function and epicardial plaque measured in the coronary artery	↑ CysSSCys/GSH associated with impaired microvascular function and greater epicardial necrotic core ↑ CysSSCys in patients with ↑ BMI and HTN
	Study of CysLTs changes during and after cardiac surgery with CPB in patients with and without COPD Ref [88]	Patients undergoing cardiac surgery with CPB: nine moderate-to-severe COPD (69 yo; 78% men) + 10 non-smoker no COPD patients (64 yo; 60% men). Urine and plasma at baseline, end of CPB, after CPB and 2 h after admission in ICU. CysLTs = LTC4 + LTD4 + LTE4	↑ urine CysLTs with time in both groups, but more evident in COPD patients; Plasma Cys LTs baseline < at admission to ICU in patients with COPD
	Assess LTE4 during and after acute coronary syndromes Ref [86]	Urine samples from 16 AMI (mean age 51 yo; 88% men); 14 UA patients (mean age 52 yo; 21% men); eight clinical CTLs (non-ischemic heart pain) (88% mean) and 10 normal CTLs (50% men) CTLs (non-evidence of coronary artery disease). Samples collected upon admission with acute chest pain and 3 days after.	LTE4 in MIA and UA at admission > CTL groups. LTE4 on admission > 3 days after UA.

↑: higher; ↓: lower; (−): negative correlation; (+): positive correlation; ACE: Angiotensin Converting Enzyme; AMI: acute myocardial infarction; BMI: body mass index; CDV: cardiovascular; CPAP: continuous positive air pressure; CPB: cardiopulmonary bypass; CTL: controls; CVE: cardiovascular event; Cys: cysteine; CysSSCys: cystine; CysSSG: cysteine-glutathione disulfide; GCF: gingival crevicular fluid; GSH: reduced glutathione; GSSG: oxidized glutathione; HTN: hypertension; ICH: intracerebral hemorrhage; ICU: intensive care unit; LT: leukotriene; LTB4: leukotriene B4; LTC4: leukotriene C4; LTD4: leukotriene D4; LTE4: leukotriene E4; min SaO2: minimal oxygen saturation; OSA: obstructive sleep apnea; SaO2, arterial oxygen saturation; T1D: type 1 diabetes; T2D: type 2 diabetes; TG: triglycerides; UA: unstable angina; yo: years old.

**Table 5:** Methodologies for mercapturomic profiling.

Analyte	Sample	Sampling and Pre-Treatment and Analyses	
**5-S-CyS-DOPA**	Serum	Sampling: blood collected into plain evacuated tubes and allowed to coagulate. Pre-treatment: commercial kit (Immundiagnostik GmbH, Bensheim, Germany). Extraction and purification on acid-washed aluminum oxide. LC-EC Ref [50]	
		Ref [51]	
		Sampling: blood collected into plain evacuated tubes and allowed to coagulate. Sample: 500 µL serum. Pre-treatment: adsorption of 5-S-cysteinyl-DOPA to alumina, washing alumina with a phosphate buffer pH 4.0 and elution of 5-S-cysteinyl-dopa with HC1O_4_. LC-EC Method from Ref [100] Ref [52,54]	
		Ref [53]	
**5-S-CyS-DA**	CSF	Sample: 70 µL CSF; Sampling: collection at 7:30 and 8:30 am, ultrafiltration into Millipore Ultrafree-MC units having a NMWC cut-off of 10,000. LC-EC Ref [68]	
		Sample: 1 mL; batch alumina extraction; LLOD 10 pmol/L, or 10 fmol per assayed mL of CSF; LC-EC Ref [67]	
**5-S-Cys-DOPA,** **5-S-Cys-DA,** **5-S-Cys- DOPAC** **5-GSH-DOPA,** **5-GSH-DA** **5-GSH-DOPAC**	Tissues from 11 different brain regions.	Homogenization and digestion with proteinase K in a digestion buffer with addition of perchloric acid 0.1 M. Centrifugation and supernatant filtration (0.22-pm Micropure separators). Adsorption of catechols from 100 µL of the filtrate to acid-washed aluminum oxide. Washing aluminum oxide with distilled water and elution of catechols with mobile phase (pH 2.7). LC Ref [74]	
**Cys-4-OHE1** **Cys-4-OHE2** **GSH-4-OHE1** **GSH-4-OHE2** **N-AcCys-4-OHE1** **N-AcCys -4-OHE2**	Spot urine	About 50 mL was collected from each participant and 1 mg/mL ascorbic acid was added to prevent oxidation of the catechol moieties. Urine samples (2 mL) were adjusted to pH 7 and then loaded into phenyl SPE pre-conditioned cartridges. Elution with methanol/10 mmM ammonium formate pH 7 (90:10) with 1% acetic acid.	LC-MS Normalization to urine creatinine Ref [58,59,60,75]
			LC-MS Normalization with depurinating estrogen DNA adducts Ref [56,57]
**Glucose-cysteine**	Urine	No extraction. GC-MS SIM of characteristic ions 632, 745, 604 and 726 m/z. Normalization by urinary creatinine Ref [4]	
**LTE4**	Urine	Urine 10 mL was stabilized by the addition of NaOH and 4-OH-TEMPO before freezing. LC purification. Radioimmunoassay LLOD 8 pg/mL. Normalization with urinary creatinine sulfate Ref [86]	
		25 mL SPE followed by LC purification. Radioimmunoassay Normalization with urinary creatinine Ref [87]	
		Spot urine. LC purification. Radioimmunoassay normalization with urinary creatinine LLOD: 6.3 pg of LTE 4 per milligram of creatinine Ref [46]	
		Ref [95] 4 mL of urine SPE LC-MS/MS in negative mode. Acquisition in MRM *m/z* 438.2 → 333.0. Normalization with urinary creatinine	
		Ref [93] Urine collection at 7:00 am. Pre-treatment SPE LC-MS/MS in negative mode. Acquisition in MRM *m/z* 438.2 → 333.0 LLOD: 10 pg/mL urine. Normalization with urinary creatinine	
		Ref [96] Overnight urine collection. Pre-treatment SPE; C-MS/MS in negative mode. Acquisition in MRM *m/z* 438.2 → 333.0 LLOD: 10 pg/mL urine. Normalization with urinary creatinine	
		Ref [99] 50 uL urine No purification of sample required. EIA Range: 7.8–1000 pg/mL. Normalization by urinary creatinine	
		Ref [94] Sample collection at 7:00 am. SPE MS/MS in negative mode. Acquisition in MRM *m/z* 438.2 → 333.0 LLOD: 10 pg/mL urine. Normalization with urinary creatinine	
Sum of **LTC4, LTD4** **LTE4**	Urine	Ref [85] Urine samples were added to 8 mL methanol containing 4-hydroxy-TEMPO (4- hydroxy-2,2,6,6-tetramethylpiperidine-N-oxyl) and ethylenediami- netetraacedic acid (EDTA) in final concentrations of 1.0 and 0.5 mM, respectively before freezing. Pre-treatment 2 mL SPE followed by LC purification. Radioimmunoassay normalization with urinary creatinine	
	Urine Saliva	1 mL saliva SPE followed by LC purification. EIA Ref [47]	
Sum of **LTC4, LTD4** **LTE4**	GSF	EIA; LLOD 7.8 pg/mL for LTC4 + LTD4 + LTE4 and 3.9 pg/mL for LTB4 Ref [92]	
**LTD4** **LTE4**	EBC	At least 1.5 mL of EBC. ELISA Ref [40]	
Sum of **LTB4, LTC4** **LTD4, LTE4**	EBC Plasma Urine	EBC (1 mL); Blood and a spot urine samples were taken between 8:00 and 12:00 a.m. Blood collected with EDTA. SPE. LC-MS/MS Ref [39]	
	Plasma Urine	SPE for plasma samples. Enzyme-linked immunosorbent assay. Urinary LTs normalized with urinary creatinine. Plasma LTs concentrations were corrected for changes in plasma protein concentration Ref [88]	
Sum of **LTA4, LTC4** **LTD4, LTE4**	Plasma	Blood collected overnight fasting in EDTA tubes. Plasma. SPE. ELISA Ref [78]	
**CysSSCys** **CySSG**	Plasma	Precipitation of potassium perchlorate with KOH/tetraborate solution followed by derivatization with dansyl chloride. Blood samples collected after overnight fasting. Blood collected into specially prepared tubes containing a preservative solution with serine, sodium heparin, BPDS, iodoacetic acid, borate and tetraborate. The supernatant was then transferred to a perchloric acid solution before freezing LC-FD Ref [101,102]	
**CysSSCys**	Plasma	Blood collected in heparin tubes and immediately placed in preservation buffer containing BPDS. The supernatant was added to ice-cold 10% perchloric acid in 10 μmol gamma-glutamylglutamate before freezing LC-FD Ref [61]	
		Blood collected in sodium heparin tubes and transferred into specially prepared tubes with preservative containing serine, sodium heparin, BPDS, iodoacetic acid and borate. Supernatant transferred into a tube containing 10% ice-cold perchloric acid and 0.2 M boric acid solution LC-FD Ref [97]	
		Blood collected in EDTA tubes. After centrifugation, butylated hydroxytoluene and salicylic acid as lipid and aqueous antioxidants were added before freezing LC-FD Ref [62]	
		Aliquots were preserved in a 5% perchloric acid solution containing iodoacetic acid (6.7 μmol/L) and boric acid (0.1 mol/L) before freezing LC-FD Ref [48]	
		Blood collected into heparin tubes and transferred into a preservative solution before freezing LC-MS Ref [103]	

2-OHE1(E2), 2-hydroxyestrone (estradiol); 4-OHE1(E2), 4-hydroxyestrone (estradiol); 5-S-Cys-DOPA: 5-S-Cysteinyl-dopa; 5-S-Cys-DA: 5-S-Cysteinyl-dopamine; BPDS: bathophenanthroline disulfonate; CSF: cerebrospinal final; Cys: cysteine; CysSSCys: cystine; CysSSG: cysteine-glutathione disulfide DA: dopamine; DOPA: L-3,4-dihydroxyphenylalanine; DOPAC 3,4-dihydroxyphenylacetic acid;; EBC: exhaled breath condensate; EIA enzyme immunoassay GC-MS: gas chromatography with mass spectrometry detection; GCF: gingival crevicular fluid; GSH: glutathione LC: liquid chromatography; LC-EC: liquid chromatography with electrochemical detection; LC-FD: liquid chromatography with fluorescence detection; LC-MS: liquid chromatography with mass spectrometry detection; LLOD: lower limit of detection; LTB4: leukotriene B4; LTC4: leukotriene C4; LTD4: leukotriene D4; LTE4: leukotriene E4; MRM multiple reaction monitoring; MS/MS tandem mass spectrometry; NAC: N-acetylcysteine.; SIM: selected ion monitoring; SPE: solid phase extraction.

## References

[1] Chambers J.C., Zhang W., Lord G.M., van der Harst P., Lawlor D.A., Sehmi J.S., Gale D.P., Wass M.N., Ahmadi K.R., Bakker S.J.L. (2010). Genetic loci influencing kidney function and chronic kidney disease. Nat. Genet..

[2] Habig W.H., Pabst M.J., Jakoby W.B. (1974). Glutathione S transferases. The first enzymatic step in mercapturic acid formation. J. Biol. Chem..

[3] Veiga-da-Cunha M., Tyteca D., Stroobant V., Courtoy P.J., Opperdoes F.R., Van Schaftingen E. (2010). Molecular identification of NAT8 as the enzyme that acetylates cysteine S-conjugates to mercapturic acids. J. Biol. Chem..

[4] Szwergold B.S. (2006). α-Thiolamines such as cysteine and cysteamine act as effective transglycating agents due to formation of irreversible thiazolidine derivatives. Med. Hypotheses.

[5] Magnay J.L., Tong J., Drangova R., Baines A.D. (2001). Production of cysteinyl-dopamine during intravenous dopamine therapy. Kidney Int..

[6] Ntimbane T., Krishnamoorthy P., Huot C., Legault L., Jacob S.V., Brunet S., Levy E., Guéraud F., Lands L.C., Comte B. (2008). Oxidative stress and cystic fibrosis-related diabetes: A pilot study in children. J. Cyst. Fibros..

[7] Feroe A.G., Attanasio R., Scinicariello F. (2016). Acrolein metabolites, diabetes and insulin resistance. Environ. Res..

[8] Ballatori N., Krance S.M., Notenboom S., Shi S., Tieu K., Hammond C.L. (2009). Glutathione dysregulation and the etiology and progression of human diseases. Biol. Chem..

[9] Hughey R.P., Rankin B.B., Elce J.S., Curthoys N.P. (1978). Specificity of a particulate rat renal peptidase and its localization along with other enzymes of mercapturic acid synthesis. Arch. Biochem. Biophys..

[10] Griffith O.W. (1981). The role of glutathione turnover in the apparent renal secretion of cystine. J. Biol. Chem..

[11] Hanigan M.H. (1998). γ-Glutamyl transpeptidase, a glutathionase: Its expression and function in carcinogenesis. Chem. Biol. Interact..

[12] Commandeur J.N., Stijntjes G.J., Vermeulen N.P. (1995). Enzymes and transport systems involved in the formation and disposition of glutathione S-conjugates. Role in bioactivation and detoxication mechanisms of xenobiotics. Pharmacol. Rev..

[13] Hinchman C.A., Rebbeor J.F., Ballatori N. (1998). Efficient hepatic uptake and concentrative biliary excretion of a mercapturic acid. Am. J. Physiol. Liver Physiol..

[14] Garnier N., Redstone G.G.J., Dahabieh M.S., Nichol J.N., del Rincon S.V., Gu Y., Bohle D.S., Sun Y., Conklin D.S., Mann K.K. (2014). The novel arsenical darinaparsin is transported by cystine importing systems. Mol. Pharmacol..

[15] Kanaoka Y., Boyce J.A. (2014). Cysteinyl leukotrienes and their receptors; emerging concepts. Allergy Asthma Immunol. Res..

[16] Townsend D.M., Deng M., Zhang L., Lapus M.G., Hanigan M.H. (2003). Metabolism of cisplatin to a nephrotoxin in proximal tubule cells. J. Am. Soc. Nephrol..

[17] Stern S.T., Bruno M.K., Horton R.A., Hill D.W., Roberts J.C., Cohen S.D. (2005). Contribution of acetaminophen-cysteine to acetaminophen nephrotoxicity II. Possible involvement of the γ-glutamyl cycle. Toxicol. Appl. Pharmacol..

[18] Lewis R.A., Austen K.F. (1984). The biologically active leukotrienes. Biosynthesis, metabolism, receptors, functions, and pharmacology. J. Clin. Investig..

[19] Rosenthal A., Pace-Asciak C.R. (1983). Potent vasoconstriction of the isolated perfused rat kidney by leukotrienes C4 and D4. Can. J. Physiol. Pharmacol..

[20] Badr K.F., Brenner B.M., Ichikawa I. (1987). Effects of leukotriene D4 on glomerular dynamics in the rat. Am. J. Physiol..

[21] Shastri S., McNeill J.R., Wilson T.W., Poduri R., Kaul C., Gopalakrishnan V. (2001). Cysteinyl leukotrienes mediate enhanced vasoconstriction to angiotensin II but not endothelin-1 in SHR. Am. J. Physiol. Hear. Circ. Physiol..

[22] Leng W., Kuo C.G., Qureshi R., Jakschik B.A. (1988). Role of leukotrienes in vascular changes in the rat mesentery and skin in anaphylaxis. J. Immunol..

[23] Guo R., Jiang J., Jing Z., Chen Y., Shi Z., Deng B. (2018). Cysteinyl leukotriene receptor 1 regulates glucose-stimulated insulin secretion (GSIS). Cell. Signal..

[24] Stern S.T., Bruno M.K., Hennig G.E., Horton R.A., Roberts J.C., Cohen S.D. (2005). Contribution of acetaminophen-cysteine to acetaminophen nephrotoxicity in CD-1 mice: I. Enhancement of acetaminophen nephrotoxicity by acetaminophen-cysteine. Toxicol. Appl. Pharmacol..

[25] Dvash E., Har-Tal M., Barak S., Meir O., Rubinstein M. (2015). Leukotriene C4 is the major trigger of stress-induced oxidative DNA damage. Nat. Commun..

[26] Salauze L., van der Velden C., Lagroye I., Veyret B., Geffard M. (2005). Circulating antibodies to cysteinyl catecholamines in amyotrophic lateral sclerosis and Parkinson’s disease patients. Amyotroph. Lateral Scler. Other Motor Neuron Disord..

[27] Carlsson H., Rappaport S.M., Törnqvist M. (2019). Protein adductomics: Methodologies for untargeted screening of adducts to serum albumin and hemoglobin in human blood samples. High-Throughput.

[28] Wang W., Ballatori N. (1998). Endogenous glutathione conjugates: Occurrence and biological functions. Pharmacol. Rev..

[29] Christ-Hazelhof E., Nugteren D.H., Van Dorp D.A. (1976). Conversions of prostaglandin endoperoxides by glutathione-S-transferases and serum albumins. Biochim. Biophys. Acta Lipids Lipid Metab..

[30] Funk C.D. (2001). Prostaglandins and leukotrienes: Advances in eicosanoid biology. Science.

[31] Haeggström J.Z., Funk C.D. (2011). Lipoxygenase and leukotriene pathways: Biochemistry, biology, and roles in disease. Chem. Rev..

[32] Di Gennaro A., Haeggström J.Z. (2012). The leukotrienes: Immune-modulating lipid mediators of disease. Adv. Immunol..

[33] Capra V., Thompson M.D., Sala A., Cole D.E., Folco G., Rovati G.E. (2007). Cysteinyl-leukotrienes and their receptors in asthma and other inflammatory diseases: Critical update and emerging trends. Med. Res. Rev..

[34] Gonçalves-Dias C., Morello J., Correia M., Coelho N., Antunes A.M.M., Macedo M.P., Monteiro E.C., Soto K., Pereira S.A. (2019). Mercapturate pathway in the tubulocentric perspective of diabetic kidney disease. Nephron.

[35] Rubinstein M., Dvash E. (2018). Leukotrienes and kidney diseases. Curr. Opin. Nephrol. Hypertens..

[36] Gelosa P., Colazzo F., Tremoli E., Sironi L., Castiglioni L. (2017). Cysteinyl leukotrienes as potential pharmacological targets for cerebral diseases. Mediat. Inflamm..

[37] Burke L., Butler C.T., Murphy A., Moran B., Gallagher W.M., O’Sullivan J., Kennedy B.N. (2016). Evaluation of cysteinyl leukotriene signaling as a therapeutic target for colorectal cancer. Front. Cell Dev. Biol..

[38] Noncommunicable Diseases. https://www.who.int/news-room/fact-sheets/detail/noncommunicable-diseases.

[39] Pelclová D., Fenclova Z., Vlcková Š., Lebedová J., Syslova K., Pecha O., Belacek J., Navrátil T., Kuzma M., Kacer P. (2012). Leukotrienes B4, C4, D4 and E4 in the exhaled breath condensate (EBC), blood and urine in patients with pneumoconiosis. Ind. Health.

[40] Celik D., Doruk S., Koseoglu H.I., Sahin S., Celikel S., Erkorkmaz U. (2013). Cysteinyl leukotrienes in exhaled breath condensate of smoking asthmatics. Clin. Chem. Lab. Med..

[41] Wennergren G. (2000). Inflammatory mediators in blood and urine. Paediatr. Respir. Rev..

[42] Gaki E., Papatheodorou G., Ischaki E., Grammenou V., Papa I., Loukides S. (2007). Leukotriene E4 in urine in patients with asthma and COPD-The effect of smoking habit. Respir. Med..

[43] Laidlaw T.M., Boyce J.A. (2012). Cysteinyl leukotriene receptors, old and new; implications for asthma. Clin. Exp. Allergy.

[44] Montuschi P. (2009). LC/MS/MS analysis of leukotriene B4 and other eicosanoids in exhaled breath condensate for assessing lung inflammation. J. Chromatogr. B.

[45] Montuschi P. (2008). Leukotrienes, antileukotrienes and asthma. Mini Rev. Med. Chem..

[46] Green S.A., Malice M.P., Tanaka W., Tozzi C.A., Reiss T.F. (2004). Increase in urinary leukotriene LTE4levels in acute asthma: Correlation with airflow limitation. Thorax.

[47] Ono E., Taniguchi M., Higashi N., Mita H., Yamaguchi H., Tatsuno S., Fukutomi Y., Tanimoto H., Sekiya K., Oshikata C. (2011). Increase in salivary cysteinyl-leukotriene concentration in patients with aspirin-intolerant asthma. Allergol. Int..

[48] Stephenson S.T., Brown L.A.S., Helms M.N., Qu H., Brown S.D., Brown M.R., Fitzpatrick A.M. (2015). Cysteine oxidation impairs systemic glucocorticoid responsiveness in children with difficult-to-treat asthma. J. Allergy Clin. Immunol..

[49] Shimbori C., Shiota N., Okunishi H. (2010). Involvement of leukotrienes in the pathogenesis of silica-induced pulmonary fibrosis in mice. Exp. Lung Res..

[50] Wimmer I., Meyer J.C., Seifert B., Dummer R., Flace A., Burg G. (1997). Prognostic value of serum 5-S-cysteinyldopa for monitoring human metastatic melanoma during immunochemotherapy. Cancer Res..

[51] Banfalvi T., Gilde K., Boldizsar M., Fejös Z., Horvath B., Liszkay G., Beczassy E., Kremmer T. (2000). Serum concentration of 5-S-cysteinyldopa in patients with melanoma. Eur. J. Clin. Investig..

[52] Wakamatsu K., Kageshita T., Furue M., Hatta N., Kiyohara Y., Nakayama J., Ono T., Saida T., Takata M., Tsuchida T. (2002). Evaluation of 5-S-cysteinyldopa as a marker of melanoma progression: 10 years’ experience. Melanoma Res..

[53] Sato S., Aoki T., Umezu K., Mori M., Hayashi M., Saito H., Kitamura K., Tsuchida A., Koyanagi Y., Yamagishi T. (2003). Rectal malignant melanoma diagnosed by N-isopropyl-p-123I-iodoamphetamine single photon emission computed tomography and 5-S-cysteinyl dopa: Report of a case. Surg. Today.

[54] Umemura H., Yamasaki O., Kaji T., Otsuka M., Asagoe K., Takata M., Iwatsuki K. (2017). Usefulness of serum 5-S-cysteinyl-dopa as a biomarker for predicting prognosis and detecting relapse in patients with advanced stage malignant melanoma. J. Dermatol..

[55] Salehi F., Dunfield L., Phillips K.P., Krewski D., Vanderhyden B.C. (2008). Risk factors for ovarian cancer: An overview with emphasis on hormonal factors. J. Toxicol. Environ. Heal. Part B Crit. Rev..

[56] Zahid M., Beseler C.L., Hall J.B., LeVan T., Cavalieri E.L., Rogan E.G. (2014). Unbalanced estrogen metabolism in ovarian cancer. Int. J. Cancer.

[57] Zahid M., Goldner W., Beseler C.L., Rogan E.G., Cavalieri E.L. (2013). Unbalanced estrogen metabolism in thyroid cancer. Int. J. Cancer.

[58] Gaikwad N.W., Yang L., Muti P., Meza J.L., Pruthi S., Ingle J.N., Rogan E.G., Cavalieri E.L. (2008). The molecular etiology of breast cancer: Evidence from biomarkers of risk. Int. J. Cancer.

[59] Gaikwad N.W., Yang L., Pruthi S., Ingle J.N., Sandhu N., Rogan E.G., Cavalieri E.L. (2009). Urine biomarkers of risk in the molecular etiology of breast cancer. Breast Cancer Basic Clin. Res..

[60] Gaikwad N.W., Yang L., Weisenburger D.D., Vose J., Beseler C., Rogan E.G., Cavalieri E.L. (2009). Urinary biomarkers suggest that estrogen-DNA adducts may play a role in the aetiology of non-Hodgkin lymphoma. Biomarkers.

[61] Jonas C.R., Puckett A.B., Jones D.P., Griffith D.P., Szeszycki E.E., Bergman G.F., Furr C.E., Tyre C., Carlson J.L., Galloway J.R. (2000). Plasma antioxidant status after high-dose chemotherapy: A randomized trial of parenteral nutrition in bone marrow transplantation patients. Am. J. Clin. Nutr..

[62] Hopkins M.H., Fedirko V., Jones D.P., Terry P.D., Bostick R.M. (2010). Antioxidant micronutrients and biomarkers of oxidative stress and inflammation in colorectal adenoma patients: Results from a randomized, controlled clinical trial. Cancer Epidemiol. Prev. Biomark..

[63] Kish S.J., Shannak K., Hornykiewicz O. (1988). Uneven pattern of dopamine loss in the striatum of patients with idiopathic parkinsons disease. Pathophysiologic and clinical implications. N. Engl. J. Med..

[64] Goldstein D.S., Jinsmaa Y., Sullivan P., Holmes C., Kopin I.J., Sharabi Y. (2016). 3,4-Dihydroxyphenylethanol (hydroxytyrosol) mitigates the increase in spontaneous oxidation of dopamine during monoamine oxidase inhibition in PC12 cells. Neurochem. Res..

[65] Kurth M.C., Adler C.H. (1998). COMT inhibition. Neurology.

[66] Fornstedt B., Brun A., Rosengren E., Carlsson A. (1989). The apparent autoxidation rate of catechols in dopamine-rich regions of human brains increases with the degree of depigmentation of substantia nigra. J. Neural Transm. Dis. Dement. Sect..

[67] Goldstein D.S., Holmes C., Sullivan P., Jinsmaa Y., Kopin I.J., Sharabi Y. (2016). Elevated cerebrospinal fluid ratios of cysteinyl-dopamine/3, 4-dihydroxyphenylacetic acid in parkinsonian synucleinopathies. Parkinsonism Relat. Disord..

[68] Cheng F.-C., Kuo J.-S., Chia L.-G., Dryhurst G. (1996). Elevated 5-S-cysteinyldopamine/homovanillic acid ratio and reduced homovanillic acid in cerebrospinal fluid: Possible markers for and potential insights into the pathoetiology of Parkinson’s disease. J. Neural Transm..

[69] Carlsson A., Fornstedt B. (1991). Catechol metabolites in the cerebrospinal fluid as possible markers in the early diagnosis of Parkinson’s disease. Neurology.

[70] Goldstein D.S., Sullivan P., Holmes C., Miller G.W., Alter S., Strong R., Mash D.C., Kopin I.J., Sharabi Y. (2013). Determinants of buildup of the toxic dopamine metabolite DOPAL in Parkinson’s disease. J. Neurochem..

[71] Riederer P., Sofic E., Rausch W.-D., Schmidt B., Reynolds G.P., Jellinger K., Youdim M.B.H. (1989). Transition metals, ferritin, glutathione, and ascorbic acid in parkinsonian brains. J. Neurochem..

[72] Jenner P., Dexter D.T., Sian J., Schapira A.H.V., Marsden C.D. (1992). Oxidative stress as a cause of nigral cell death in Parkinson’s disease and incidental lewy body disease. Ann. Neurol..

[73] Spencer J.P.E., Jenner P., Halliwell B. (1995). Superoxide-dependent depletion of reduced glutathione by L-DOPA and dopamine. Relevance to parkinson’s disease. Neuroreport.

[74] Spencer J.P.E., Jenner P., Daniel S.E., Lees A.J., Marsden D.C., Halliwell B. (1998). Conjugates of catecholamines with cysteine and GSH in Parkinson’s disease: Possible mechanisms of formation involving reactive oxygen species. J. Neurochem..

[75] Gaikwad N.W., Murman D., Beseler C.L., Zahid M., Rogan E.G., Cavalieri E.L. (2011). Imbalanced estrogen metabolism in the brain: Possible relevance to the etiology of Parkinson’s disease. Biomarkers.

[76] Pardo C.A., Vargas D.L., Zimmerman A.W. (2005). Immunity, neuroglia and neuroinflammation in autism. Int. Rev. Psychiatry.

[77] Li X., Chauhan A., Sheikh A.M., Patil S., Chauhan V., Li X.M., Ji L., Brown T., Malik M. (2009). Elevated immune response in the brain of autistic patients. J. Neuroimmunol..

[78] Qasem H., Al-Ayadhi L., El-Ansary A. (2016). Cysteinyl leukotriene correlated with 8-isoprostane levels as predictive biomarkers for sensory dysfunction in autism. Lipids Health Dis..

[79] Janicka M., Kot-Wasik A., Kot J., Namieśnik J. (2010). Isoprostanes-biomarkers of lipid peroxidation: Their utility in evaluating oxidative stress and analysis. Int. J. Mol. Sci..

[80] Simmet T., Seregi A., Hertting G. (1987). Formation of sulphidopeptide-leukotrienes in brain tissue of spontaneously convulsing gerbils. Neuropharmacology.

[81] Kiwak K.J., Moskowitz M.A., Levine L. (2009). Leukotriene production in gerbil brain after ischemic insult, subarachnoid hemorrhage, and concussive injury. J. Neurosurg..

[82] Moskowitz M., Kiwak K., Hekimian K., Levine L. (2006). Synthesis of compounds with properties of leukotrienes C4 and D4 in gerbil brains after ischemia and reperfusion. Science.

[83] Winking M., Lausberg G., Simmet T., Piscol K., Klinger M., Brock M. (1992). Malignancy-dependent formation of cysteinyl-leukotrienes in human brain tumor tissues and its detection in urine. Neurosurgical Standards, Cerebral Aneurysms, Malignant Gliomas.

[84] Bittl J.A., Pfeffer M.A., Lewis R.A., Mehrotra M.M., Corey E.J., Austen K.F. (1985). Mechanism of the negative inotropic action of leukotrienes C4 and D4 on isolated rat heart. Cardiovasc. Res..

[85] Winking M., Deinsberger W., Joedicke A., Boeker D.K. (1998). Cysteinyl-leukotriene levels in intracerebral hemorrhage: An edema-promoting factor?. Cerebrovasc. Dis..

[86] Carry M., Korley V., Willerson J.T., Weigelt L., Ford-Hutchinson A.W., Tagari P. (1992). Increased urinary leukotriene excretion in patients with cardiac ischemia: In vivo evidence for 5-lipoxygenase activation. Circulation.

[87] Allen S.P., Sampson A.P., Piper P.J., Chester A.H., Ohri S.K., Yacoub M.H. (1993). Enhanced excretion of urinary leukotriene E4 in coronary artery disease and after coronary artery bypass surgery. Coron. Artery Dis..

[88] de Prost N., El-Karak C., Avila M., Ichinose F., Melo M.F.V. (2011). Changes in cysteinyl leukotrienes during and after cardiac surgery with cardiopulmonary bypass in patients with and without chronic obstructive pulmonary disease. J. Thorac. Cardiovasc. Surg..

[89] Söder P.Ö., Söder B., Nowak J., Jogestrand T. (2005). Early carotid atherosclerosis in subjects with periodontal diseases. Stroke.

[90] Grau A.J., Becher H., Ziegler C.M., Lichy C., Buggle F., Kaiser C., Lutz R., Bültmann S., Preusch M., Dörfer C.E. (2004). Periodontal disease as a risk factor for ischemic stroke. Stroke.

[91] Persson G.R., Ohlsson O., Pettersson T., Renvert S. (2003). Chronic periodontitis, a significant relationship with acute myocardial infarction. Eur. Heart J..

[92] Bäck M., Airila-Månsson S., Jogestrand T., Söder B., Söder P.-Ö. (2007). Increased leukotriene concentrations in gingival crevicular fluid from subjects with periodontal disease and atherosclerosis. Atherosclerosis.

[93] Stanke-Labesque F., Bä M., Lefebvre B., Tamisier R., Baguet J.-P., Arnol N., Lé P., Pé J.-L., Grenoble F., Stockholm S. (2009). Increased urinary leukotriene E4 excretion in obstructive sleep apnea: Effects of obesity and hypoxia. J. Allergy Clin. Immunol..

[94] Gautier-Veyret E., Bäck M., Arnaud C., Belaïdi E., Tamisier R., Lévy P., Arnol N., Perrin M., Pépin J.-L., Stanke-Labesque F. (2018). Cysteinyl-leukotriene pathway as a new therapeutic target for the treatment of atherosclerosis related to obstructive sleep apnea syndrome. Pharmacol. Res..

[95] Hardy G., Boizel R., Bessard J., Cracowski J.L., Bessard G., Halimi S., Stanke-Labesque F. (2005). Urinary leukotriene E4 excretion is increased in type 1 diabetic patients: A quantification by liquid chromatography-tandem mass spectrometry. Prostaglandins Other Lipid Mediat..

[96] Boizel R., Bruttmann G., Benhamou P.Y., Halimi S., Stanke-Labesque F. (2010). Regulation of oxidative stress and inflammation by glycaemic control: Evidence for reversible activation of the 5-lipoxygenase pathway in type 1, but not in type 2 diabetes. Diabetologia.

[97] Dhawan S.S., Eshtehardi P., McDaniel M.C., Fike L.V., Jones D.P., Quyyumi A.A., Samady H. (2011). The role of plasma aminothiols in the prediction of coronary microvascular dysfunction and plaque vulnerability. Atherosclerosis.

[98] Ashfaq S., Abramson J.L., Jones D.P., Rhodes S.D., Weintraub W.S., Hooper W.C., Vaccarino V., Harrison D.G., Quyyumi A.A. (2006). The relationship between plasma levels of oxidized and reduced thiols and early atherosclerosis in healthy adults. J. Am. Coll. Cardiol..

[99] Rafnsson A., Bäck M. (2013). Urinary leukotriene E4 is associated with renal function but not with endothelial function in type 2 diabetes. Dis. Mark..

[100] Wakamatsu K., Ito S. (1994). Improved HPLC determination of 5-S-cysteinyldopa in serum. Clin. Chem..

[101] Jones D.P., Carlson J.L., Mody V.C., Cai J., Lynn M.J., Sternberg P. (2000). Redox state of glutathione in human plasma. Free Radic. Biol. Med..

[102] Ashfaq S., Abramson J.L., Jones D.P., Rhodes S.D., Weintraub W.S., Hooper W.C., Vaccarino V., Alexander R.W., Harrison D.G., Quyyumi A.A. (2008). Endothelial function and aminothiol biomarkers of oxidative stress in healthy adults. Hypertension.

[103] Patel R.S., Ghasemzadeh N., Eapen D.J., Sher S., Arshad S., Ko Y., Veledar E., Samady H., Zafari A.M., Sperling L. (2016). Novel biomarker of oxidative stress is associated with risk of death in patients with coronary artery disease. Circulation.

[104] Mathias P.I., B’Hymer C. (2016). Mercapturic acids: Recent advances in their determination by liquid chromatography/mass spectrometry and their use in toxicant metabolism studies and in occupational and environmental exposure studies. Biomarkers.

[105] Nunes J., Charneira C., Morello J., Rodrigues J., Pereira S.A., Antunes A.M.M. (2019). Mass Spectrometry-Based Methodologies for Targeted and Untargeted Identification of Protein Covalent Adducts (Adductomics): Current status and challenges. High-Throughput.

